# Biobanks for Genomics and Genomics for Biobanks

**DOI:** 10.1002/cfg.333

**Published:** 2003-12

**Authors:** Anne Cambon-Thomsen, Pascal Ducournau, Pierre-Antoine Gourraud, David Pontille

**Affiliations:** 1 Inserm U 558, Epidémiologie et Analyses en Santé Publique: Risques Maladies Chroniques et Handicaps Faculté de Médecine 37 Allées Jules Guesde Toulouse cedex F-31073 France; 2 FRE2674 Centre Interdisciplinaire de Recherches Urbaines et Sociologiques (CIRUS) Université Toulouse le Mirail (Toulouse II) Maison de la Recherche 5 Allée Antonio Machado Toulouse cedex 1 31058 France; 3 UMR5044 Centre d'Étude et de Recherche Techniques, Organizations Pouvoirs (CERTOP) Université Toulouse le Mirail (Toulouse II) Maison de la Recherche, 5 Allée Antonio Machado Toulouse cedex 1 31058 France

## Abstract

Biobanks include biological samples and attached databases. Human biobanks occur in research, technological development and medical activities. Population genomics is highly dependent on the availability of large biobanks. Ethical issues must be considered: protecting the rights of those people whose samples or data are in biobanks (information, autonomy, confidentiality, protection of private life), assuring the non-commercial use of human body elements and the optimal use of samples and data. They balance other issues, such as protecting the rights of researchers and companies, allowing long-term use of biobanks while detailed information on future uses is not available. At the level of populations, the traditional form of informed consent is challenged. Other dimensions relate to the rights of a group as such, in addition to individual rights. Conditions of return of results and/or benefit to a population need to be defined. With ‘large-scale biobanking’ a marked trend in genomics, new societal dimensions appear, regarding communication, debate, regulation, societal control and valorization of such large biobanks. Exploring how genomics can help health sector biobanks to become more rationally constituted and exploited is an interesting perspective. For example, evaluating how genomic approaches can help in optimizing haematopoietic stem cell donor registries using new markers and high-throughput techniques to increase immunogenetic variability in such registries is a challenge currently being addressed. Ethical issues in such contexts are important, as not only individual decisions or projects are concerned, but also national policies in the international arena and organization of democratic debate about science, medicine and society.


**Comparative and Functional Genomics**
*Comp Funct Genom* 2003; **4**: 628–634.
Published online in Wiley InterScience (www.interscience.wiley.com). **DOI:** 10.1002/cfg.333

**Conference Review**

### Biobanks for genomics and genomics for biobanks

Anne Cambon-Thomsen[1]*, Pascal Ducournau[1,2], Pierre-Antoine Gourraud[1] and David Pontille[1,3]

[1] *Inserm U 558, Epidémiologie et Analyses en Santé Publique: Risques, Maladies Chroniques et Handicaps, Faculté de Médecine, 37 Allées J Guesde, 31073 Toulouse cedex, France*
[2] *FRE2674 Centre Interdisciplinaire de Recherches Urbaines et Sociologiques (CIRUS), Université Toulouse le Mirail (Toulouse II), Maison de la Recherche, 5 Allée Antonio Machado, 31058 Toulouse cedex 1, France*
[3] *UMR5044 Centre d'Étude et de Recherche Techniques, Organizations, Pouvoirs (CERTOP), Université Toulouse le Mirail (Toulouse II), Maison de la Recherche, 5 Allée Antonio Machado, 31058 Toulouse cedex 1, France*

**Correspondence to: Anne Cambon-Thomsen, Inserm U 558, Epidémiologie et Analyses en Santé Publique: Risques, Maladies Chroniques et Handicaps, Faculté de Médecine, 37 Allées Jules Guesde, F-31073 Toulouse cedex, France. E-mail: cambon@cict.fr*

### Abstract

**Biobanks include biological samples and attached databases. Human biobanks occur in research, technological development and medical activities. Population genomics is highly dependent on the availability of large biobanks. Ethical issues must be considered: protecting the rights of those people whose samples or data are in biobanks (information, autonomy, confidentiality, protection of private life), assuring the non-commercial use of human body elements and the optimal use of samples and data. They balance other issues, such as protecting the rights of researchers and companies, allowing long-term use of biobanks while detailed information on future uses is not available. At the level of populations, the traditional form of informed consent is challenged. Other dimensions relate to the rights of a group as such, in addition to individual rights. Conditions of return of results and/or benefit to a population need to be defined. With 'large-scale biobanking' a marked trend in genomics, new societal dimensions appear, regarding communication, debate, regulation, societal control and valorization of such large biobanks. Exploring how genomics can help health sector biobanks to become more rationally constituted and exploited is an interesting perspective. For example, evaluating how genomic approaches can help in optimizing haematopoietic stem cell donor registries using new markers and high-throughput techniques to increase immunogenetic variability in such registries is a challenge currently being addressed. Ethical issues in such contexts are important, as not only individual decisions or projects are concerned, but also national policies in the international arena and organization of democratic debate about science, medicine and society. Copyright © 2003 John Wiley & Sons, Ltd.**

Received: 21 August 2003
Revised: 25 August 2003
Accepted: 10 September 2003

**Keywords: human biobanks; ethics; genetic databases; population databases; societal issues of genomics; public health genomics; haematopoietic stem cell donor registries**

### Introduction

Because collections of samples offer multiple long-term scientific interests, the use of human biological samples as biobanks occurs in a variety of situations, from research and technological development to medical diagnosis and therapeutic activities. The genome-based sciences have generated numerous needs for population studies [36]. The population dimension of genomics is highly dependent on the availability of large biobanks adapted to the scale of the studies envisaged. Managing such large biobanks has become technically feasible, but it is important to understand the general context of

Copyright © 2003 John Wiley & Sons, Ltd.

biobanks in order to identify ethical issues that relate to them [10,15].

### Biobanking prior to the genomic era

Historically, human genetic bio-collections have mainly been motivated by the study of diseases (most commonly of rare diseases) and have been more family-based than population-based [17]. Population-based collections have long existed in the context of genetic anthropology and the history of world populations, but these are usually academic and modestly sized. Large epidemiological collections of biological samples have rarely been turned towards genetic data. Genetic epidemiologists have often claimed that knowing the population frequencies of the polymorphisms studied in diseases and families is an important parameter for genetic analyses. However, it has been difficult both to get relevant large populations and to gather financial support for results that appeared very theoretical and distant from economical interests.

### Consideration of issues relating to biobanking

Issues in biobanking, which were first seen as technical issues by professionals, then as ethical concerns (also mainly by professionals), are increasingly being considered by regulatory authorities and at the political level [42]. It is not only a matter of good technical conservation of samples, optimal database construction, and means of individual respect or protection, but also a matter of scientific political decision at the level of national resource exploitation. In fact, not only scientists but also regulatory authorities and administrative entities are considering this topic within their scope [42]. Conferences specifically focusing on multidisciplinary issues in biobanking have been organized, and not only by scientists [1–5]. Several European countries have issued specific legislations regarding biobanking (e.g. Iceland, Sweden, France and Estonia) and several International or National Bioethics Committees are preparing, or have already given, opinions on this subject [30]. 'Biobanks for genomics' has become a key issue at the societal level.

### What is a biobank?

The concept of biobank includes the biological samples themselves (of different kinds corresponding to variable conservation conditions, but all may be sources of nucleic acids), the attached databases, and a certain level of openness, availability and exchange for different kinds of studies. General categories of samples are family samples, or unrelated individuals with various degrees of identification of the person (identified, identifiable, anonymized or anonymous) and different kinds of information attached to the sample (personal, medical) *a priori* or *a posteriori* (e.g. resulting from a lab test). In addition to information attached to the sample, there are types of information relating to a group of people that are also important (e.g. frequencies of markers in a population, general information about a sampled population) [10,17].

### Why are biobanks important today?

Several factors are now converging to motivate the development of large population-based collections. The number of available polymorphic markers is rapidly increasing (especially SNPs) and automated molecular techniques and bioinformatics tools are at hand for mass screening from small amounts of sample. Polymorphisms are often related to function and may play a role in the development of common diseases, and in the responses of individuals to treatment [33]. They may also give clues for the development of new therapeutic molecules. Thus, data on polymorphisms of all sorts become of primary importance, not only to academic or medical geneticists but also to pharmaceutical companies and the biotechnology industry [6,35,37]. In this context, the tendency has been to try to constitute large population collections in various countries, with European examples in Iceland, Estonia, Latvia, Sweden and the UK [8,36,38]. Private funds, sometimes in combination with public funding, are supporting those collections that become part of the national resources. Official recognition and identification of biobanking activity, as well as financial sustainability, are required. There is a need for education in biobanking and for the production of guidelines on the quality of collections, including the management of ethical issues. The activity of biobanker becomes a profession and

Copyright © 2003 John Wiley & Sons, Ltd. 

requires certain modes of certification or accreditation [32].

### General ethical tensions in the context of biobanking

A schematic representation of the various dimensions and fields of application of biobanking is shown in Figure 1, which underlines the gap between the interests and individual expectations of donors and the present development of biobanking in various contexts. The tensions encountered are often a mixture of technical and quality issues and of philosophical/societal dimensions. Analyses of such issues have been detailed elsewhere [12,15,16,25,45] but the following can be underlined:

- How to protect the rights of people whose samples and data are in the biobanks (autonomy, confidentiality and protection of private life), whilst allowing and encouraging research [7]?
- How to ensure the non-commercial use of human body elements, whilst allowing the use of the samples in the development of commercial products [6,37]? Samples themselves may be directly involved in drug development, with the extension of various biotherapies (cell therapies, gene therapies, etc.): when does a sample become a product, a reagent?
- How to inform donors correctly when one does not know what possible developments there will be over time? The dimensions of informed consent are complex in this matter [22]. Between express consent, enlarged consent and presumed consent, there is place for other dimensions, such as a form of solidarity through sharing information for a common good [18].
- How to reconcile the logic that considers samples as part of body elements in some contexts, with that which considers samples as sources of data, more than as a body part, in other contexts [30]? When does a genomic sample become data?
- How to ensure that there is a maximum quality of sample conservation and management, whilst allowing easy access, without complication?
- How to optimally and openly use the samples for the rapid progress of knowledge, whilst protecting the rights of priority of the researchers who constituted the collection, balancing the need for recognition of this activity and the interests of companies [21]? This is not trivial, and one could think of methods of recognition of the effort and quality of biobanking, while opening biobank access, by setting parameters such as biobank impact factor (BIF) [11].
- How to ensure long-term financial sustainability whilst the use and the interests involved may vary over time? This was one of the points of concern that came out of a survey on biobanks in several European countries [32].
- How to avoid potential unwanted consequences, in terms of stigmatization of specific groups or misuse of results?

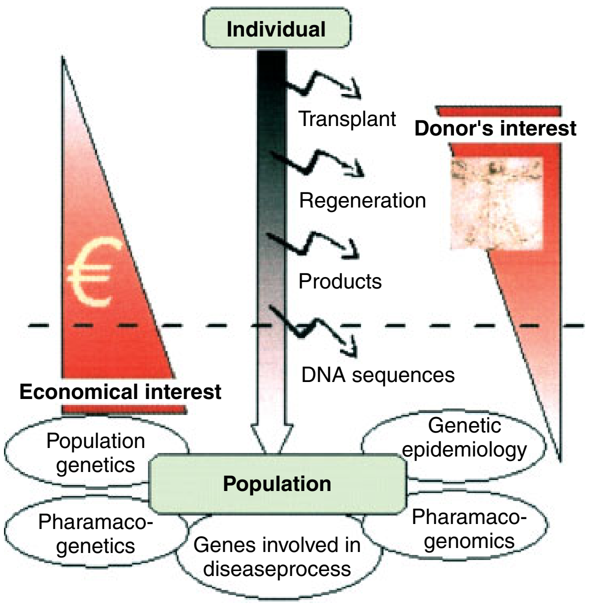

**Figure 1.** From small-scale to large-scale biobanking: various dimensions and issues

### Specific features of population genomics related to ethics

There are specific issues when considering working at the population level:

- Access to a healthy population, not always in relation to medical purposes or in a medical research context, e.g. for constructing a random control sample, or for genomic anthropology studies. Statistical considerations may be difficult to combine with individual freedom: can

Copyright © 2003 John Wiley & Sons, Ltd. 

sampling be truly 'random' and representative and completely respect the voluntary basis and informed consent?
- Different cultural contexts, which influence in particular the extent of 'non-commercial use' definition and acceptance; it may be the rule in certain contexts and not in others.
- Individual informed consent vs. group consent. The traditional role of informed consent as an expression and protection of the autonomy of the person is challenged [50]. Generally, it was focused on individual consent. This adjusts adequately to biobank projects that concern a few individuals or families. The concept of group consent has been introduced for small communities or populations in a different cultural context and was formulated as a protocol as early as 1993 [31]. The collective dimension that it implies may vary according to cultural context and the size of the population and can be met through a group or collective consent. But it generates the question, 'Who speaks for the group?'.
- Working at the international level, with various regulatory texts in the various countries [4,44]. This large variation in texts addresses the question of the status of the biological samples, which may vary according to the context: physical attribute of a person, object of the research, object of the commercial activities. This anthropological flexibility plays on two registers: that of the proprietary world, the category of 'having', that allows exchanges, patents, various property rights; and that of 'being', with the notion of patrimonial value (individual, familial, ethnical, national, etc.).
- If biobank use leads to the generation of population categorizations, how to prevent misuse of those categorizations and how to avoid social constructions compromising the scientific activity of categorization [24,40]?
- The kind of results to be returned and notions of benefit sharing may be thought of at the population level, rather than at the individual level. How to define and ensure just return to the populations who donated the samples?
- Sample management and database organization are issues that combine technical and ethical considerations. Of particular importance are the rules of access and level of openness for exchanges of samples and data between interested parties (research groups, companies, health services). The recent interest of industrial groups in collected population samples, especially in the context of pharmacogenetics studies [35,37], was mainly unplanned at the time of sampling for existing collections.

A very valuable effort has been made in Canada, where reference documents on ethical principles for research in genetics, and a consent model (with various options for different kinds of genetic studies), have been proposed, as well as a statement of principles on the ethical conduct of human research involving populations [46,47] (**http://www.rmga.qc.ca**).

### Does large-scale biobanking change the ethical issues involved?

A comparison with the developments in physics is relevant, as this discipline faced a change in scale years ago. As in physics, genomics corresponds to a change of scale, an industrial style work organization with the use of large instrumental platforms and the involvement of large consortia [28,43]. Such changes of scale and global approaches are general trends of present genome sciences; this dimension is covered by the suffix '–omics' in genomics, proteomics, etc. However, the use of biological material and especially that of human origin gives a specific dimension to genomics that is not shared by physics. 'Large-scale biobanking' induces changes in the practical management of ethical issues that have so far been addressed in the context of smaller biobanks, while the ethical principles themselves do not change. In addition, new societal dimensions are appearing, when a whole population (or a large part of it) is affected, regarding communication, debate, societal control and valorization of such large biobanks, and integration of this activity in the society at large rather than only in the scientific community. With this change of scale and scope in biobanking, it makes sense to talk about 'biobank-omics' [13].

Building large-scale biobanks necessarily involves rethinking the ethical and legal frame of personal protection and its translation into action. For example, the collective dimension of consent is difficult to achieve and may be not adapted conceptually for large populations. It may rather be approached by a collective debate before the start

Copyright © 2003 John Wiley & Sons, Ltd.

of a project and before individual consents are pursued: then a person can take a decision that does not involve only him/herself as an individual, but takes into account the dimensions underlined through this collective debate. One of the challenges is thus to elaborate a debate in the frame of an 'ethics discourse' [29], in order to avoid particular interests dominating the fair collective accord.

The case of large populations is of special relevance in Europe, where several ambitious projects have been launched with special attention to ethical issues [8], but that generate controversy [9]. But in fact, the large population-based collections are only part of the present picture of biobanking in Europe, as was shown by a survey that collected data from 147 institutions (mostly public or private non-profit) in six EU countries using questionnaires and interviews [32]. This activity is increasing in all countries, but most collections are small; only a few are very large. One of the issues in setting standards is to work out how, on the one hand, the new large-scale applications can best be organized in respect to ethical principles [18,39], without, on the other hand, preventing the continuation and use of the large variety of biobanks that have proved their value over many years [48]. Harmonizing the framework for consent forms, further use and gene ownership, and constructing a European view on benefit sharing all appear to be important.

### Biobanks are important for genomics, but is genomics important for biobanks?

Can genomics help to improve the organization or the performance of biobanks constituted and used in the context of health services? Usually researchers or companies in the field of genomics see the potential uses of biobanks for meeting their own objectives, and the application of genomics approaches for improving existing biobanks is less often considered. Biobanks in the form of tissue collections, or haematopoietic stem cell donor registries for transplantation [19,49] exist in the health sector. Their organization and use could be dramatically changed using genomics approaches. The EU project 'MADO' (for Marrow Donors) (**www.euromado.org**, [41]) is one example. Its main objective is the optimization of haematopoietic stem cell donor registries in Europe, by evaluating a number of strategies in order to increase the adequacy of the files of potential volunteer bone marrow donors to meet the needs of patients. Compatibility between donor and recipient for the major histocompatibility complex (HLA) is a key factor for the outcome of grafts. In 70% of cases, an HLA-compatible familial donor is not available and an unrelated donor has to be found [27]. As a matter of fact, although more than 8 million HLA-typed potential donors are registered worldwide, only certain patients can actually be transplanted; this is partly due to the difficulty of getting an HLA-compatible donor for each of them, given the high polymorphism of this system [20,23,34]. One of the problems is that some HLA types are more frequent than others and become over-represented in the registries, while rare ones remain absent. The interest is focused especially on how to increase the immunogenetic heterogeneity of such registries in a cost-efficient way. The project aims at finding ways of increasing the proportion of donors presenting rare HLA combinations, in order to reduce the inequality of chances of patients to find a donor and to avoid spending resources on donors whose HLA type is already in excess. The approach is based on an evaluation of genomic techniques with a high potential for automation and of large-scale practice. The concept explored is a molecular filter, that should allow the screening of potential donors, at low cost, for the likely presence of frequent HLA combinations. Could typing for markers in the HLA region predict frequent or rare HLA types without performing the expensive HLA typing, and thus become a useful tool to choose who to then completely HLA-type? The financial resources would accordingly be allocated in priority to HLA-type new potential donors with a highly probable non-frequent HLA combination in order to maximize the chances of qualitatively enriching the registries. The molecular approaches for such a filter include different markers (microsatellites, SNPs) and different techniques (high-throughput genotyping by sequencing, or by mass spectrometry, etc.). This approach is combined with other approaches aimed at elucidating sociological models underlying the recruitment and ethical requirements. This should allow the definition of conditions to access a diversified population and the most suitable ways to recruit donors that will secure a regular increase of the registry diversity in full respect of transparent donor information. The above approaches and the economic evaluation will be considered together

Copyright © 2003 John Wiley & Sons, Ltd. 

to design integrated scenarios for both recruitment and typing strategies. First results show that the HLA predictive value of microsatellites could be of interest for this application [14,26]. Genomics could lead to important reorganization of biobanking activity in this health sector, and thus all dimensions, from organizational and economical ones to ethical and sociological ones must be considered. Such use of genomics for helping biobanks in the health sector, combining technical, ethical and societal issues, certainly is an interesting perspective.

### Conclusion

Biobanking is crucial for genomics. It is a lively and growing activity, which has been carried out in numerous institutions for a considerable time. Its rather loose organization, when on a small scale, is not adapted to forthcoming large-scale projects. The era of large-scale biobanking introduces changes in the way ethical issues must be dealt with, especially with the involvement of large populations as such. The new biobanking activities are triggering ethical dimensions regarding the further use of existing collections. Exploring how genomics can help biobanks in the health sector to become more rationally constituted and exploited is an open perspective. The consideration of ethical issues in such contexts is of primary importance, as it is related not only to individual decisions or projects, but also to national policies in the international arena and to the organization of democratic debate about science, medicine and society.

### Acknowledgements

This work was supported by Genopole Toulouse Midi-Pyrénées and the EU MADO contract (QLG7-CT-2001-00065), and is partly based on results acquired during the EU project EUROGENBANK (BIOTECH EU Contract No. BIO4-CT98-0570).

### References

1. 2001. Conference: Ethics and Biomedical Research — The Process of Balancing Benefits and Risks, 11–12 June 2001, Umeå, Sweden. **http://www.eu2001.se/education/eng/docs/umea_progr_en.asp**.
2. 2002. Conference: Conference on Biobanks, 12–13 September 2002, Uppsala, Sweden. **http://www-conference.slu.se/biobanks/index.htm**.
3. 2002. Conference: Ethics in Research and Science. Situation and Perspectives in the Candidate Countries to the European Union, 17–19 March 2002, Bratislava, Slovak Republic.
4. 2002. Conference: Third International DNA Sampling Conference. 5–8 September 2002, Montreal, Canada.
5. 2003. Conference: Biobanks for Health Workshop, 28–31 January 2003, Oslo, Norway. **http://www.fhi.no/hvaskjer/biobanks_workshop.html**.
6. Anderlik M. 2003. Commercial biobanks and genetic research: ethical and legal issues. *Am J Pharmacogenom* **3**: 203–215.
7. Anderlik MR, Rothstein MA. 2001. Privacy and confidentiality of genetic information: what rules for the new science? *Ann Rev Genom Hum Genet* **2**: 401–433.
8. Austin MA, Harding S, McElroy C. 2003. Genebanks: a comparison of eight proposed international genetic databases. *Commun Genet* **6**: 37–45.
9. Barbour V. 2003. UK Biobank: a project in search of a protocol? *Lancet* **361**: 1734–1738.
10. Blatt RJ. 2000. Banking biological collections: data warehousing, data mining, and data dilemmas in genomics and global health policy. *Commun Genet* **3**: 204–211.
11. Cambon-Thomsen A. 2003. Assessing the impact of biobanks. *Nature Genet* **34**: 25–26.
12. Cambon-Thomsen A. 2003. [Biological sample banks: ethical and legal aspects. General introduction]. *Rev Epidemiol Sante Publ* **51**: 99.
13. Cambon-Thomsen A. 2003. La bioéthique à l'échelle de la 'biobanquomique'. *L'Observatoire de la Génétique (on-line)* **10**: **http://www.ircm.qc.ca/bioethique/obsgenetique/cadrages/cadr2003/c_no2010_2003/c_no2010_2003_2002.html**.
14. Cambon-Thomsen A, Foissac A, Fort M, *et al*. 2003. Microsatellites in the HLA region: association with HLA Class I and Class II genes, and strategies for bone marrow donor registries. In *HLA 2002: Immunobiology of the Human MHC*, vol 2, Hansen J, Dupont B (eds). IHWC Press: Seattle (in press).
15. Cambon-Thomsen A, Rial-Sebbag E. 2003. [Ethical aspects of biological sample banks]. *Rev Epidemiol Sante Publ* **51**: 101–110.
16. Cambon-Thomsen A, Rial-Sebbag E, Duchier J. 2003. [Ethical and legal aspects of biological sample banks: synthesis, practical questions and proposals]. *Rev Epidemiol Sante Publ* **51**: 121–126.
17. Caze de Montgolfier S. 2002. Collecte, stockage et utilisation des produits du corps humain dans le cadre des recherches en génétique: état des lieux historique, éthique et juridique; analyse des pratiques au sein des biothèques. Thèse d'Université, Université René Descartes, Paris V, Paris.
18. Chadwick R, Berg K. 2001. Solidarity and equity: new ethical frameworks for genetic databases. *Nature Rev Genet* **2**: 318–321.
19. Confer DL. 1997. Unrelated marrow donor registries. *Curr Opin Hematol* **4**: 408–412.
20. Confer DL. 2001. The National Marrow Donor Program. Meeting the needs of the medically underserved. *Cancer* **91**: 274–278.
21. Dahlquist G. 2002. [Biobanks must be used rationally. Stop quarreling about the ownership!]. *Lakartidningen* **99**: 3484–3485.
22. Deschenes M, Cardinal G, Knoppers BM, Glass KC. 2001. Human genetic research, DNA banking and consent: a question of 'form'? *Clin Genet* **59**: 221–239.

Copyright © 2003 John Wiley & Sons, Ltd. 

23. Dodson KL, Coppo PA, Confer DL. 1999. The National Marrow Donor Program: improving access to hematopoietic stem cell transplantation. *Clin Transpl* 121–127.
24. Editorial. 2000. Census, race and science. *Nature Genet* **24**: 97–98.
25. ESHG. 2000. Data storage and DNA banking for biomedical research: informed consent, confidentiality, quality issues, ownership, return of benefits. A professional perspective. European Society of Human Genetics. **http://www.eshg.org/ESHG DNA banking bckgrnd.pdf**.
26. Foissac A, Fort M, Clayton J, *et al*. 2001. Microsatellites in the HLA region: HLA prediction and strategies for bone marrow donor registries. *Transplant Proc* **33**: 491–492.
27. Gahrton G, van Rood JJ, Oudshoorn M. 2003. World Marrow Donor Association (WMDA). The World Marrow Donor Association (WMDA): its goals and activities. *Bone Marrow Transpl* **32**: 121–124.
28. Galison P, Hevly B. 1992. *Big Science: The Growth of Large Scale-Research*. Stanford University Press: Stanford.
29. Habermas J. 1991. *Erläuterungen zur Diskursethik*: Suhrkamp Verlag: Frankfurt.
30. Hansson MG, Levin M. 2003. *Biobanks as Resources for Health*. Uppsala University: Uppsala, 276.
31. HGDP. 1999.. Model ethical protocol for collecting DNA samples. Stanford, USA: Human Genome Diversity Project-Morrison Institute; 1999 **http://www.stanford.edu/group/morrison/hgdp/protocol.html**.
32. Hirtzlin I, Dubreuil C, Preaubert N, *et al*. 2003. An empirical survey on biobanking of human genetic material and data in six EU countries. *Eur J Hum Genet* **11**: 475–488.
33. Hodgson SV, Popat S. 2003. Polymorphic sequence variants in medicine: a challenge and an opportunity. *Clin Med* **3**: 260–264.
34. Hurley C. 2002. HLA diversity: detection and impact on unrelated hematopoietic stem cell donor characterization and selection. *Int J Hematol* **76**: 152–154.
35. Issa AM. 2000. Ethical considerations in clinical pharmacogenomics research. *Trends Pharmacol Sci* **21**: 247–249.
36. Kaiser J. 2002. Biobanks. Population databases boom, from Iceland to the U.S. *Science* **298**: 1158–1161.
37. Kaiser J. 2002. Biobanks. Private biobanks spark ethical concerns. *Science* **298**: 1160.
38. Kaiser J. 2003. Genomic medicine. African-American population biobank proposed. *Science* **300**: 1485.
39. Kaye J, Martin P. 2000. Safeguards for research using large scale DNA collections. *Br Med J* **321**: 1146–1149.
40. Lee S, Mountain J, Koenig BA. 2001. The meanings of race in the new genomics: implications for health disparities research. *Yale J Health Policy* Law Ethics: 33–75.
41. MADO: Optimization of typing policies for European MArrow DOnor Registries : socio-economic evaluation of molecular techniques and recruitment strategies. QLG7-CT-2001–00065 2002. In: 2003. EU R&D fifth framework programme. Quality of Life and Management of Living Resources Programme. RTD projects of Generic Nature, 13. **www.euromado.org**.
42. OECD 2001. Biological Resource Centres: Underpinning the Future of Life. Sciences and Biotechnology; OECD Code 932001041E1. **http://oecdpublications.gfi-nb.com/cgi-bin/OECDBookShop.storefront/EN/product/932001041E1**.
43. Price DJ. 1963. *Little Science, Big Science*. Columbia University Press: New York.
44. Reymond MA, Steinert R, Escourrou J, Fourtanier G. 2002. Ethical, legal and economic issues raised by the use of human tissue in postgenomic research. *Dig Dis* **20**: 257–265.
45. Rial-Sebbag E. 2003. Aspects juridiques des banques d'échantillons biologiques. *Rev Epidemiol Sante Publ* **51**: 111–120.
46. Statement of principles: human genome research. 2000. Quebec Network of Applied Genetic Medicine (RMGA). **www.rmga.qc.ca**.
47. Statement on the ethical conduct of genetic research involving populations. 2002. Quebec Network of applied genetic Medicine (RMGA). **www.rmga.qc.ca**.
48. Stegmayr B, Asplund K. 2003. [Genetic research on blood samples stored for years in biobanks. Most people are willing to provide informed consent]. *Lakartidningen* **100**: 618–620.
49. van Rood JJ, Schipper RF, Bakker JN, *et al*. 1998. Bone marrow donors worldwide and cord blood stem cell transplantation. *Bone Marrow Transplant* **22**: S19–21.
50. Wolpe PR. 1998. The triumph of autonomy in American bioethics: a sociological view. In *Bioethics and Society*, DeVries R, Sudebi J (eds). Prentice Hall: Upper Saddle River; 38–59.

Copyright © 2003 John Wiley & Sons, Ltd. 
